# Localizing Heritage: Archaeologies of the Recent Past in Postwar Jaffna, Sri Lanka

**DOI:** 10.1007/s41636-026-00636-0

**Published:** 2026-07-07

**Authors:** Gill Juleff, Thamotharampillai Sanathanan, Ruth Young, John P. Cooper, Emma Dwyer, Adrián Oyaneder

**Affiliations:** 1 Humanities and Social Sciences, Cornwall, Peter Lanyon Building, University of Exeter Penryn Campus, Penryn, Cornwall, TR10 9FE United Kingdom; 2 Department of Fine Arts, University of Jaffna, Post Box 57, Ramanathan Road, Thirunelvely, Jaffna, Sri Lanka; 3 School of Heritage and Culture, University of Leicester, University Road, Leicester, LE1 7RH United Kingdom; 4 Institute of Arab and Islamic Studies, University of Exeter, Stocker Road, Exeter, EX4 4ND United Kingdom; 5 Museum of London Archaeology (MOLA), 46 Eagle Wharf Road, London, N1 7ED United Kingdom; 6 Department of Anthropology, Universidad de Tarapacá, 18 de Septiembre 2222, Arica, Chile

**Keywords:** Jaffna, Sri Lanka, historical archaeology, nonelite heritage, community archaeology

## Abstract

The role of archaeology in divisive nationalism, colonial supremacism, and the marginalization of subaltern groups is increasingly evident globally. Such legacies are visible in Sri Lanka, not least in its north and east, where many regard archaeology as either irrelevant or as a weapon directed against them. We outline the genesis of a UK-Sri Lankan project that responds to this situation and focus on a workshop held in Jaffna with University of Jaffna partners that tested the potential for archaeology and heritage to foster affirmation of identity and sense of place in communities recovering from conflict. The initiative, which was composed of collaborative activity not requiring specialist training, brought together people of diverse disciplinary backgrounds to identify and record local sites. While archaeologies of conflict surfaced, the country’s civil war was not the primary focus. The work considered how the material fabric of place and the understanding and memory it evokes are forms of archaeology and heritage, and might be practiced for social good.

## Introduction

The mistrust of archaeology and archaeologists working in northern and eastern Sri Lanka is the painful consequence of the division between the island’s majority Sinhalese and minority Tamil communities over many decades, from colonial times through independence and civil war to the present. There exists an established bias in favor of the elite, monumental archaeology and heritage of the majority that largely marginalizes other forms of archaeological heritage. This bias is both the result of the major divisions in Sri Lankan society and a contributing factor to them.

Against this backdrop of mistrust and lack of connection with archaeology and heritage, our work in Jaffna, the town and eponymous peninsula at the northern tip of the island, has two distinct but interdependent aims. The first is to explore and record the heritages and archaeologies of the everyday—local and nonelite—of the recent and contemporary past, and the second is to use this as a means of potentially detoxifying archaeology and resituating it as a community-engaged activity that contributes positively to postwar social regeneration through the centering of local identity, sense of place, and belonging. We use the phrase “localizing heritage” to describe the process of consciously directing our focus toward an archaeological heritage that is quotidian and rooted in the locality, thus allowing communities to explore heritage values that resonate more closely with their lived experiences. By localizing heritage we hope to offer an alternative to national and international heritage that favors commodification and a hierarchy of prestige, ultimately epitomized by UNESCO World Heritage status. Essential to this is a collaboration between UK-based archaeologists and academic colleagues in Jaffna of more-diverse disciplinary backgrounds, including fine arts, economics, drama, and social sciences, to develop methods and practices that can be participatory, inclusive, and meaningful for local communities.

We begin by summarizing Sri Lanka’s current ethnic and religious makeup and the key events and issues of recent political and social history, providing context to the country’s current approaches to archaeological heritage and giving examples of how archaeological heritage can be distorted to damaging effect. To that end, it is necessary to review the place archaeology has occupied in the sociopolitical life of Sri Lanka, even though our aim is to put these aside in favor of a different framework.

We then outline the development of our collaboration as a response to these issues, leading to our first workshop in Jaffna in 2022. We used the workshop to explore the potential for archaeology to foster postwar affirmation of identity, expressed as a sense of place and belonging within a locality and landscape. We saw it also as an opportunity to demonstrate archaeological fieldwork as an activity that does not require specialist training and brings people together in common purpose. While our conceptual and practical approaches in localizing heritage may well be useful in other parts of the world facing similar challenges, we are fully aware that this type of work is particularly context dependent.

Finally, we consider the challenges we faced, our achievements, and some of the issues raised, and reflect on the impact and effectiveness of archaeology for and with non-archaeologists in the circumstances of contemporary Jaffna. We conclude with directions for developing future research and collaboration, particularly concerning the currently popular concept of “cocreating” archaeological heritage projects and engaging local communities.

## Political Background

**Fig. 1:**
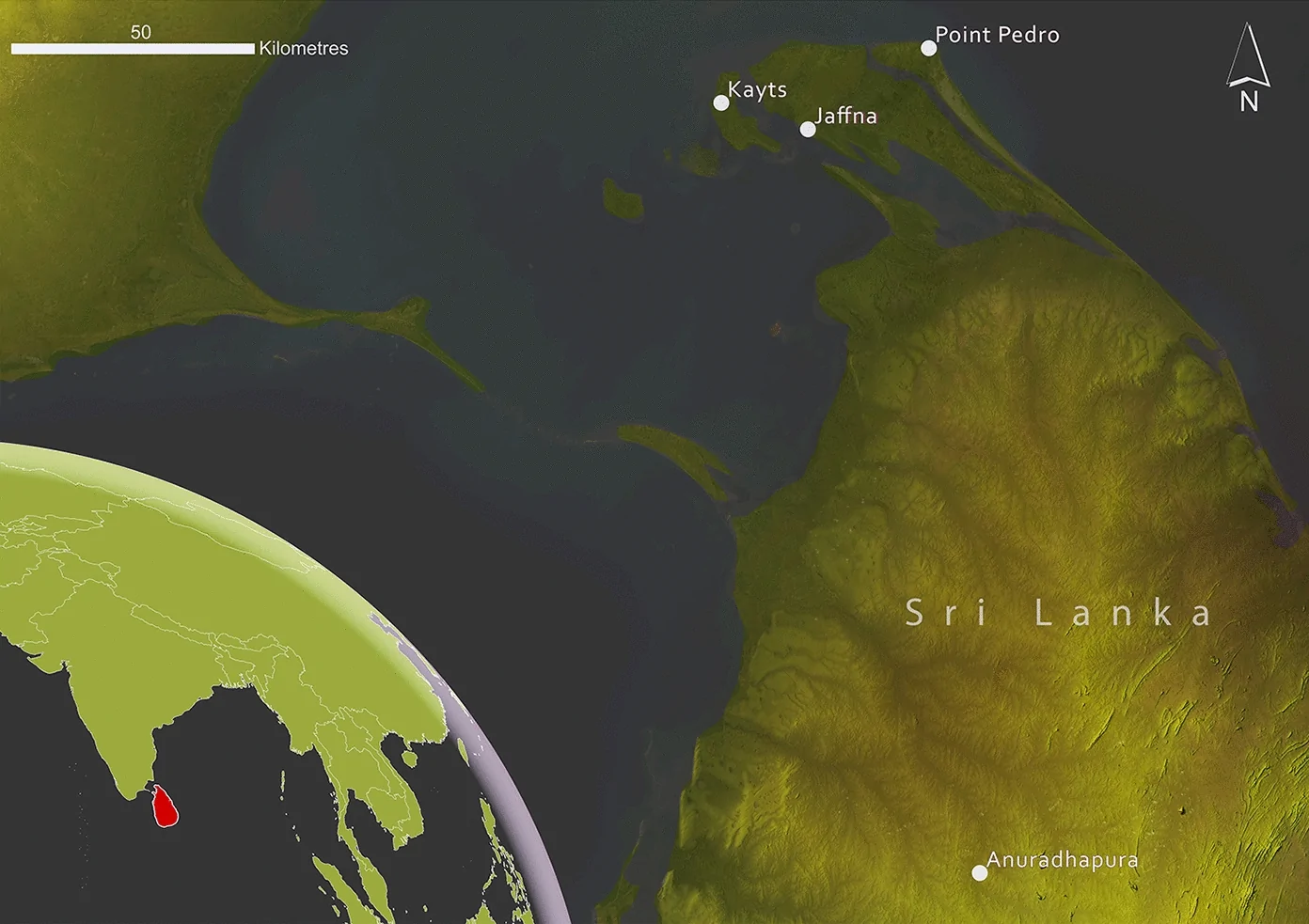
The Jaffna Peninsula in relation to India and northern Sri Lanka, with the locations of fieldwork. (Map by authors, 2023.)

The British took over the coastal areas of Sri Lanka from the Dutch in 1796 and in 1815 conquered the interior Kingdom of Kandy, uniting the island as a single polity (K. de Silva 2005; Bandarage 2008). Realizing, however, that the island was in fact a very mixed, socially heterogeneous place, the British instituted a racial classification and used this as the basis for sociopolitical representation within the imperial regime. This formalization of difference ultimately resulted in the reification and codification of the most numerous groups, the Sinhalese and Tamils, alongside a persistent narrative of historical, almost timeless, conflict between the two (Nissan and Stirrat 1990; Bandarage 2008; Haputhanthri et al. 2024). Sri Lanka’s population today is composed of a largely Sinhalese Buddhist majority community (ca. 75%) that primarily occupies the central and southern areas and a substantial Sri Lankan Tamil community of Hindus and Christians (ca. 11%) in the north and east, with Jaffna as its heartland (Fig. 1) (Northwestern: Pritzker School of Law 2021). The island also has a significant Muslim community and other smaller minorities (Hussein 2016). Indian cultural influences are also in play, with the Sinhalese tracing their ancestry to the Sanskrit-speaking north and the Tamils aligning with the Dravidian south.

Alongside the political and legal structures created by British administrators, the contemporaneous construction by European scholars of the Aryan language groups and concomitant racial theories helped support the emergent primacy of the Sinhalese, linked to (Aryan) North India (Gunawardena 1990). Similarly, the work of early British archaeologists on the island drew heavily on the *Mahavamsa*, the dominant chronicle of kingship, which was composed by Buddhist monks in the 5th century C.E. (Bechert 1978; Scheible 2016). Colonial readings of the *Mahavamsa* in the 19th century consciously linked ancient cities to the Sinhalese. For example, James E. Tennent (1859:401), the colonial secretary of Ceylon from 1846 to 1850, argued that the Sinhalese were “the Gangetic race, who were the earliest colonists,” and thus responsible for all that was civilized, while “Malabars” (Tamils), were the very opposite and indeed aspired to “impoverish and debase.” Thus, as Hartemann (2022:81) argues of colonial archaeology more generally, British archaeology in Sri Lanka not only served the colonial order, but also fed “its structural and historical inequalities.”

The emergence of Buddhist nationalism in the second half of the 19th century, exemplified in the work and writings of Angarika Dharmapala, then became a challenge to aspects of colonial rule (Gamage 2021). Buddhist institutions gained in power, culminating in the 1972 constitution’s recognition of Buddhism as the state religion (Gamage 2021). The *Mahavamsa* also played a role in the Buddhist nationalist movement because it not only offered a wealth of material closely linked to place, but also provided an historical legitimation of Buddhists as Sri Lanka’s rightful owners and occupants. Burgeoning nationalism was also enacted in the resettling of the “Dry Zone,” the island’s most extensive central and eastern ecozone, from the mid-20th century on, with the *Mahavamsa* tank, temple, and village idealized model being promoted by politicians alongside the “reawakening” of the ancient kingdoms of Anuradhapura and Polonnaruwa (Spencer 1990:10).

In 1948 Sri Lanka gained independence and entered a period of political adjustment, largely characterized by division and escalating unrest due to the increasing polarization between the dominant groups.^1^ For example, a 1956 law made Sinhala the sole official language of the country (Spencer 1990:2). This was followed by Tamil expressions of concern around settlement in the Eastern Province, articulated as “a form of state-sponsored encroachment of ‘Tamil areas’ by the Sinhalese” (Bandarage 2008:47). Divisions between the two communities deepened, driven by political schisms and employment and development decisions that disadvantaged Tamils. By the mid-1970s an embryonic Tamil separatist movement had grown visible (Spencer 1990; Bandarage 2008; Hashim 2013). Spencer (1990:9) characterizes the postindependence country as a state lacking both unity and universally relevant identities and heritages: only those of the Sinhala Buddhist community were nationally recognized and promulgated.

The Sri Lankan civil war erupted in 1984 and continued until 2009. While the origins, trajectories, and outcomes of the conflict have been examined in detail elsewhere (Spencer 1990; Wickramasinghe 2006; Thiranagama 2011; Hashim 2013), we provide a brief background summary here. The Liberation Tigers of Tamil Ealam (LTTE) emerged as the dominant military group with demands for a self-governing state in the north and east of the island, and began a campaign of insurgency against the Sri Lankan military and government. The LTTE was designated a terrorist group by more than 30 countries, although some have rescinded this designation since 2009 (Canton 2021). The conflict was fought across the Jaffna Peninsula, the Eastern Province, and in the sparsely inhabited Vanni area of scrub jungle that separates Jaffna from the rest of the island. Although there were also LTTE attacks and political assassinations in the capital, Colombo, and parts of the south (Bandarage 2008), the conflict had greatest impact on civilians in the majority-Tamil areas. Between 80,000 and 100,000 people were killed over 30 years (Hashim 2013:4).

The impacts of the war are still felt today, from the trauma of bereavement, dispossession, and displacement to the destruction of infrastructure and underinvestment in regeneration. Some Sri Lankans assert that their country may be postwar but is not yet post-conflict. As a collective concerned with building a team of Jaffna colleagues alongside UK archaeologists, we had few illusions as to the continuing legacy of war and its significance to our work.

### Heritage and the Role of Archaeology

We now turn to a closer appraisal of the role of archaeological heritage, specifically within the broader context of conflict. Both communities claim long-standing settlement on the island, drawing selectively on archaeology and history to support their claims (Nissan and Stirrat 1990). Since independence, the dominant discourse has been one of modern Sinhalese Buddhists as the rightful possessors of the island, with the Tamil Hindus as their adversaries and aspirant usurpers (Spencer 1990:3; K. de Silva 2005; Indrapala 2005).

Archaeology, history, and heritage have played a major role in the construction of this discourse. As Spencer (1990:3) notes: “The war which has been fought between the armed Tamil separatists and the Sinhala-dominated government has been accompanied by rhetorical wars fought over archaeological sites, place-name etymologies, and the interpretation of ancient inscriptions.” Archaeology, history, and heritage, especially in popular forms, have been powerful actors in the creation of Sri Lanka’s divisive social and political structures. Yet history and archaeology also document that the population of Sri Lanka is complex and multifaceted, and not the simple religio-ethnic dichotomy commonly presented (Deraniyagala 1996:3; K. de Silva 2005). For example, the early reach of Buddhism deep into peninsular India from its North Indian origins gave rise to a significant Tamil Buddhist community in the 1st millennium C.E. Thus the presence of Buddhist remains does not automatically equate to Sinhalese occupation, as is assumed today and used to support claims of Sinhalese primacy (Nissan and Stirrat 1990:23; Indrapala 2005).

#### Divisive Histories and Archaeologies

Difference, tension, and conflict between the Sinhalese and Tamils has found support in selective accounts of the island’s history. For example, the mythologized historical rivalry between the 2nd-century B.C.E. Sinhalese King Dutugemunu and the Tamil Prince Elara from the southern Indian Chola dynasty for control of the ancient capital of Anuradhapura is now a key historical narrative (Nissan and Stirrat 1990; Dewasiri 2013). Their battle is presented today as pivotal in the Sinhalese struggle for ascendency and unification of the island. Such reductive narratives have become received wisdom, permeating education, the arts, and popular culture (Karunaratne 2021). It has, for example, found its way into the *Encyclopædia Britannica*, which reports that Dutugemunu is “remembered as a national hero for temporarily ending the domination of the Indian Tamil Hindus over the Sinhalese” (Britannica 2024). Yet this mythologization chooses to ignore the complexity of multiethnic, multireligious Sri Lanka in the 2nd century B.C.E.—and long before and after—when, as K. de Silva (2005:17) points out, there was no simple binary.

The site of Kantharodai on the Jaffna Peninsula encapsulates many of the persistent issues of Tamil-Sinhalese tension. The site, a habitation mound and series of small Buddhist stupas (visible today as the core of a heritage site open to the public and promoted to international tourists) is generally described as an early historical urban center and monastic site, and has been the subject of numerous excavations since 1917 (Ragupathy 1987:57; Murphy et al. 2018). Below the Buddhist layers, earlier megalithic phases have been identified, with material culture indicating contact with South India back to the 5th century B.C.E., according to radiocarbon estimates (Deraniyagala 1992:730; Indrapala 2005:69,75) and more-recent dating (Murphy et al. 2018:98–99). Material culture evidence includes Lakshmi plaque coins with a “mother goddess” on one side and a “swastik [*sic*] cum anchor symbol,” believed to be a Tamil Brahmi inscription, on the other (Ragupathy 1987:58–59). Interpretations of Kantharodai thus range from it being a Buddhist site, supporting Sinhalese Buddhist claims of early settlement in the northern areas of Sri Lanka, to a Tamil Hindu site predating the arrival of Buddhism (Nissan and Stirrat 1990:23; Indrapala 2005; Harris 2019). Initial investigations at Kantharodai by the Sri Lankan civil servant Paul Pieris in 1917, alongside work by the British civil servant J. P. Lewis, explicitly present the Buddhist remains here as solid evidence to support the Sinhala Buddhists as the island’s first occupants (Harris 2019:6). The interpretation of the site as Buddhist owes a great deal to these assertions, which have remained unchallenged by later excavations by the government’s Department of Archaeology. Ragupathy (1987: 57) sums up local antipathy towards the department thus: “[Its] motives … in excavating Kantarōtai were not scientific and academic. The Department was interested only in highlighting the Buddhist monuments and in reconstructing them.”

These two examples demonstrate that tacit assumptions of polarized identities from the 1st millennium B.C.E./C.E. onward are articulated today through authorized heritage. They obscure a reality of long-term coexistence and a nuanced social fabric of ethnicities, religions, economies, histories, and communities reflecting a history of external cultural and trade connections spanning India, Arabia, East Africa, Southeast Asia, and colonial Europe that have been adapted and distilled into multiple, distinct local identities (K. de Silva 2005; Haputhanthri and S. de Silva 2021).

#### Archaeology and Heritage Today

Heritage is today a significant component of Sri Lanka’s economy and national identity. The island has five UNESCO cultural World Heritage sites from the 1st millennium C.E., and these underpin cultural tourism (unesco: World Heritage Convention 2026). Internationally renowned sites, such as Anuradhapura, Sigiriya, Polonnaruwa, and Dambulla, are elite, monumental, and linked to state-backed narratives—the “official heritage” of Spencer (1990:3) and “authorised heritage discourse” of Smith (2006, 2015)—that support the preeminence of royal dynasties. This focus on elite sites of the Early and Middle Historic periods linked to Sinhalese and Buddhist power offers a largely monocultural version of the past that eclipses more-diverse heritages (Haputhanthri et al. 2024).

All archaeological sites in Sri Lanka fall under the control of the government Department of Archaeology, which is charged with the maintenance and protection of the country’s archaeological heritage (Department of Archaeology, Sri Lanka 2026). The department was established in 1890 under British rule, building on the work by colonial officers exploring and recording ancient remains. The greatest attention was given to the ancient cities of Anuradhapura and Polonnaruwa with their visible structures, and the deciphering of a wealth of rock-cut inscriptions. After independence, the model of the department continued unchanged, albeit under the directorship of Sri Lankan archaeologists. Today the department is responsible for over 250,000 sites. That many of these are living religious sites of pilgrimage and worship is a significant added complication in their management and presentation (Wijesuriya 2003).

In the 1980s, the Sri Lankan government established the Central Cultural Fund to conserve and manage the six “exceptional” sites of the newly-coined “Cultural Triangle of Sri Lanka,” composed of Polonnaruwa, Sigiriya, Dambulla, the Abhayagiri and Jetavana monastic complexes at Anuradhapura, and the later Temple of the Tooth Relic complex of Kandy, under the auspices of UNESCO (Silva 1993). This financial and physical investment signaled the importance of archaeological heritage in Sri Lanka and formed the foundation of a highly visible tourist industry that has transformed the island into a cultural destination on the international map and generated substantial income. All the sites in the Cultural Triangle are Sinhalese Buddhist, publicly and internationally reinforcing Sri Lankan heritage as monocultural.

Against this background, feelings of anger and alienation in Sri Lanka’s north are today exacerbated by the government’s triumphalist and highly visible memorialization of sites associated with the war and the redeployment of military and naval personnel to undertake historic-site restorations.^2^ This presence is evident in the restoration of structures, such as the Dutch forts at Pooneryn and Arippu, where interpretation boards give credit to military and naval personnel for their work. While the deployment of military personnel to restore sites may have been well-intentioned, their presence in practice reasserts the dominance of the victorious regime (Harris 2019). Memorialization in its various manifestations is central to identity and heritage creation in most cultures and periods worldwide. Yet in Sri Lanka’s north and east, far-reaching prohibitions on the memorialization of Tamil war losses remain a source of bitter resentment. In addition, the unchecked and provocative appropriation by Buddhist clergy and nationalist activists of ritual and religious sites, including very minor and forgotten locations, using archaeological finds to justify claims, represents further politicization of heritage (Pieris 2019; Frerks 2023). Memorialization of the LTTE (including monuments and commemoration events) has been extremely controversial and challenging (McCargo and Seneratne 2020; Tamil Guardian 2024); this is an issue in its own right and an area we plan to explore further in our wider project.

### Project Development

Against this backdrop, we the authors are developing a project focusing on localized archaeologies of the recent past of the Jaffna Peninsula that allows us to explore the rich and diverse evidence of the everyday and is firmly rooted in place and locality. Our work applies historical archaeology approaches in two broad domains. The first is the built environment, including administrative and religious structures, buildings associated with European contact and economic activity, and the domestic and mundane. The second is artisanship, particularly focused on landscape and coastal activity. This later historical archaeological heritage forms the physical fabric of village and town environments today and merges with the social memory and material culture of numerous communities. The reality of the long coexistence of Muslim, Hindu, Christian, and Buddhist faith communities and multiple ethnicities outside the state-endorsed narrative of division is overdue for recognition as shared heritage (Haputhanthri and S. de Silva 2021).

The field workshop of April 2022 discussed below was the result not of an opportunistic intervention by foreign researchers, but of several years of dialogue among partners. It is useful to reflect on the genesis of the project because it charts an important and careful process of contact and trust building. For two of us (Juleff and Young), our first step was to draw on personal knowledge of and experience with Sri Lanka and South Asian archaeology in order to debate, between ourselves and with Sri Lankan colleagues, the current state and position of the discipline in the context of an officially promoted environment of post-conflict reconciliation and peacebuilding. Our first scoping visits of 2016–2017 allowed us to meet colleagues in universities, NGOs, civil-society organizations (CSOs), and other settings from the far south to the far north of the island. What struck us was the contrast between a continuing and broad acceptance in the south that the core function of archaeology is to objectively illuminate the material record of ancient history—albeit by excavating elite sites of the Early and Middle Historic periods—and, in the north, the mistrust of archaeology as an instrument of government used to reinforce the supremacy of the majority.

Through a review of our experiences during these initial visits we identified three areas where taking different approaches might make a new contribution. Firstly, the archaeology of the last 500 years, reflecting periods of external contact, colonial occupation, and independence, is both rich and largely unexplored, not least because the sub-discipline of historical archaeology has made little impact here (Young 2019). In the north of the island the material fabric of the last 500 years remains relatively well-preserved, notwithstanding the war damage to many structures. Secondly, with official focus firmly on the elite monumental sites of the Early Historic period, the archaeology of everyday life in any period other than the prehistoric rarely features in the local repertoire. Thirdly, from talking with (particularly younger) academics and practitioners in archaeology, history, museology, heritage, the arts, and social sciences it was clear that there is an appetite to challenge old tropes and redefine the meaning and place of archaeology and heritage in contemporary Sri Lankan society.

During these discussions the most probing engagements came from those outside the disciplines of archaeology and history, and included creative artists; activists challenging postwar reconstruction policies; those in CSOs supporting resettlement, regeneration, and peace-building activities; and even those in hospitality working to rebalance the touristic promotion of the island. An example of this is the work of one of our authors and project coleader (Thamotharampillai Sanathanan), an established artist and academic whose creative output maps memory and loss in the north. His 2011 project, “The Incomplete Thombu,” culminated in an art book that records the floor plans of houses and buildings lost during the war and draws from the memories of those who lived in them, combined with semitransparent overlays akin to archaeological field records, together with artistic responses to the stories told by those remembering (Sanathanan 2011). A *thombu* is a public land registry and was a format used across the island during the Dutch period. In an early conversation, Sanathanan commented that he had not thought of his work as a form of archaeology. Another example is the work of Historical Dialogues, a Colombo-based project that explored postwar memorialization and diverse historical narratives through place-based walks, talks, and online events. The project has developed into the Collective for Historical Dialogue and Memory (CHDM 2024). It became clear that the intersectional space between archaeology and other disciplines offered opportunities for new viewpoints on archaeology and heritage.

The process of scoping helped us to formulate a project framework and nurture relationships with academics and non-academics. We feel it is important to highlight this stage of project development, particularly if there is to be input by multiple stakeholders and a genuine process of codevelopment. We (Juleff and Young in the early inception of the project, and all the project team and current authors in subsequent engagement) are very aware of the strong pull of colonial knowledge production in places such as Sri Lanka and the impacts this has on individuals and institutions, as well as its role in the conditions that resulted in civil war. The current climate within academic research and funding, dominated as it as by the Global North, pays inadequate attention to the value of in-person engagement in partnership building and favors the use of remote contact through e-mail, shared digital resources, and digital meeting platforms (accelerated during and since the COVID-19 pandemic). Our experience has been that colleagues do not feel like equal partners in these situations due to time and language differences and without the advantage of reliable, institution-supported technology and Internet connections.

Early dialogue led us to a first exploratory two-day event in Jaffna in 2019 under the auspices of the Walking Heritage into Future Cities (WHFC) Project (Haputhanthri 2019). Working with Heritage Walk Calcutta (now Immersive Trails [2026]), an academic-led creative social enterprise based in Kolkata, India, we brought together a self-selected group of students, activists, members of the public, academics, and practitioners to discuss the meaning of heritage and to experience the discovery and sharing of heritage at street level in Jaffna town. It was at this workshop that the negative impact of top-down state-authorized heritage narratives and the visible contribution of archaeology to these narratives was emphatically and passionately expressed by young people who had grown up through war and who only knew their city in conflict (Juleff 2019; Haputhanthri et al. 2024). The degree of mistrust of archaeology and archaeologists was palpable. Through challenging, often heated, but open discussions, we worked toward a better-shared appreciation of what archaeology might be (particularly as a methodological tool for investigating the material past) and what heritage can do (as a means of acknowledging different pasts and valuing knowledge of past places, experiences, people, and events). Especially effective in overcoming biases was the physicality of walking together through the city and identifying material features that could be claimed as local heritage.

With this experience in mind, we were, after the hiatus of the COVID-19 pandemic, able to begin to put ideas into action. With Jaffna colleagues we developed a practical strategy for a five-day workshop aiming to spend time in the field to get to know each other, learn about individual interests and specialisms, explore locations, and share archaeological methods. The workshop was therefore a key moment in the development of the project and the project team. The authorship of this article, weighted as it is toward scholars based in the United Kingdom (Universities of Exeter and Leicester, Museum of London Archaeology), reflects the state of the project at that time. As we describe below, one result of the 2022 workshop was a significant diversification of the endeavor and move away from the dichotomy of external (and foreign) specialist vs. local recipient.

### Jaffna Historical Archaeology Workshop April 2022

The urban/semi-urban nature of Jaffna’s settlement patterns and the ubiquity of the sea guided our workshop planning. Peninsular Jaffna supports a current population of ca. 600,000, a decline compared to a prewar (1981) census figure of over 750,000 (State of Sri Lankan Cities 2022). The majority live in Jaffna town and its many satellite towns and villages. Much of the peninsula is composed of lagoonal and low-lying land, with several large islands connected by causeways and regular ferry routes (Fig. 1). These islands suffered significant depopulation during the civil war, leaving many remains of former settlements and past economic activity.

At this stage our objective was to pilot collaborative fieldwork and apply nondestructive archaeological methods; we were not pursuing specific research questions. As a framework we adopted a landscape-biography approach (Marsh and Jones 2014; Kolen et al. 2015; van der Schriek 2019). To experience different landscape types, we selected three areas based on discussions with local colleagues. Each area was treated as a transect that could be walked, with features within it being observed, discussed, and recorded as a transect narrative, so that a biography for each began to emerge. Walking methods have been used in both archaeology and heritage studies, particularly around memory, place, and material culture (Anderson 2004; Whitehead et al. 2021). Rather than being constrained by a systematic and standardized archaeological field-walking methodology and to optimize the data captured, we adopted an opportunistic and responsive style of fieldwork. The transect narrative might include geographical coordinates; notes, sketches, and photographs of topographical or vegetational features; moveable and fixed material culture; and informal human interactions. Features and structures encountered, such as buildings, traditional boats, and maritime structures, provided opportunities for more-sustained specialist recording, including the creation of measured plans of buildings and photogrammetry of boats. Ultimately, data will be compiled using digital mapping techniques so that places can be analyzed through multiple layers of reporting.

As a group with mixed experience and skills (archaeologists, historians, geographers, economists, sociologists, and creative humanities practitioners), we also wanted to pay attention to the dynamics of our group interactions and how this affected what was deemed important and thus recorded.

The workshop’s Jaffna team was drawn from university staff and students, but also included independent researchers and practitioners associated with the Faculty of Arts at the University of Jaffna, all self-motivated. It is important to note that, while the staff of the university are predominantly local to Jaffna, their student cohort is drawn from across the island and includes young people from Tamil-speaking communities in the east and the hill country, as well as Tamil- and Sinhala-speaking communities in Colombo and other urban centers. Thus, the fieldwork areas were familiar to some and unfamiliar to others, bringing a variety of perspectives. The program was composed of a combination of days and half days in the field, with time in the classroom to review and reflect on fieldwork. The group quickly developed a habit of information sharing and lively open discussion that generated valuable feedback for future project development.

The heart of the workshop was the time spent in the field, and here we give a flavor of three different walk days. These accounts are anecdotal, yet give an insight into the diversity of features encountered and the dynamics of the process of field recording and dialogue. The data gathered during these walks form a component of our project archive that is not reported here. Instead, the narrative style of these vignettes gives an opportunity to consider the methodological trajectory of the approach we have adopted. As part of the cocreation process it was and remains important, given the sociopolitical environment in which the project is evolving, that we proactively review and self-reflect on our actions and impacts.

It should be noted here that participants in the workshop (and who have become project team members) were self-selecting, whether staff and students from the University of Jaffna or responding to local publicity. All participants declared they had an interest in archaeology and heritage, which ranged from critique of official heritages to interest in local and personal heritage, and, while they were prepared to discuss and analyze heritage critically, they were united in wanting to learn more and find new ways to engage with local archaeology and heritage. The participants thus did not represent all segments of society, and those with more-entrenched views were disinclined to take part. In consequence, the adversarial atmosphere experienced at the opening of the 2019 event did not recur in 2022. While several of the 2019 attendees returned to participate in 2022, the understandings we had reached in 2019 were maintained collectively, and the consensus was to put aside grievances with the national discourse, at least temporarily, in favor of promoting local interests. Finding ways to engage with disenfranchised marginalized communities and their views are priorities for the wider ongoing project and will be discussed in future publications.

### Three Walks

#### The Old Railway in Jaffna Town

**Fig. 2:**
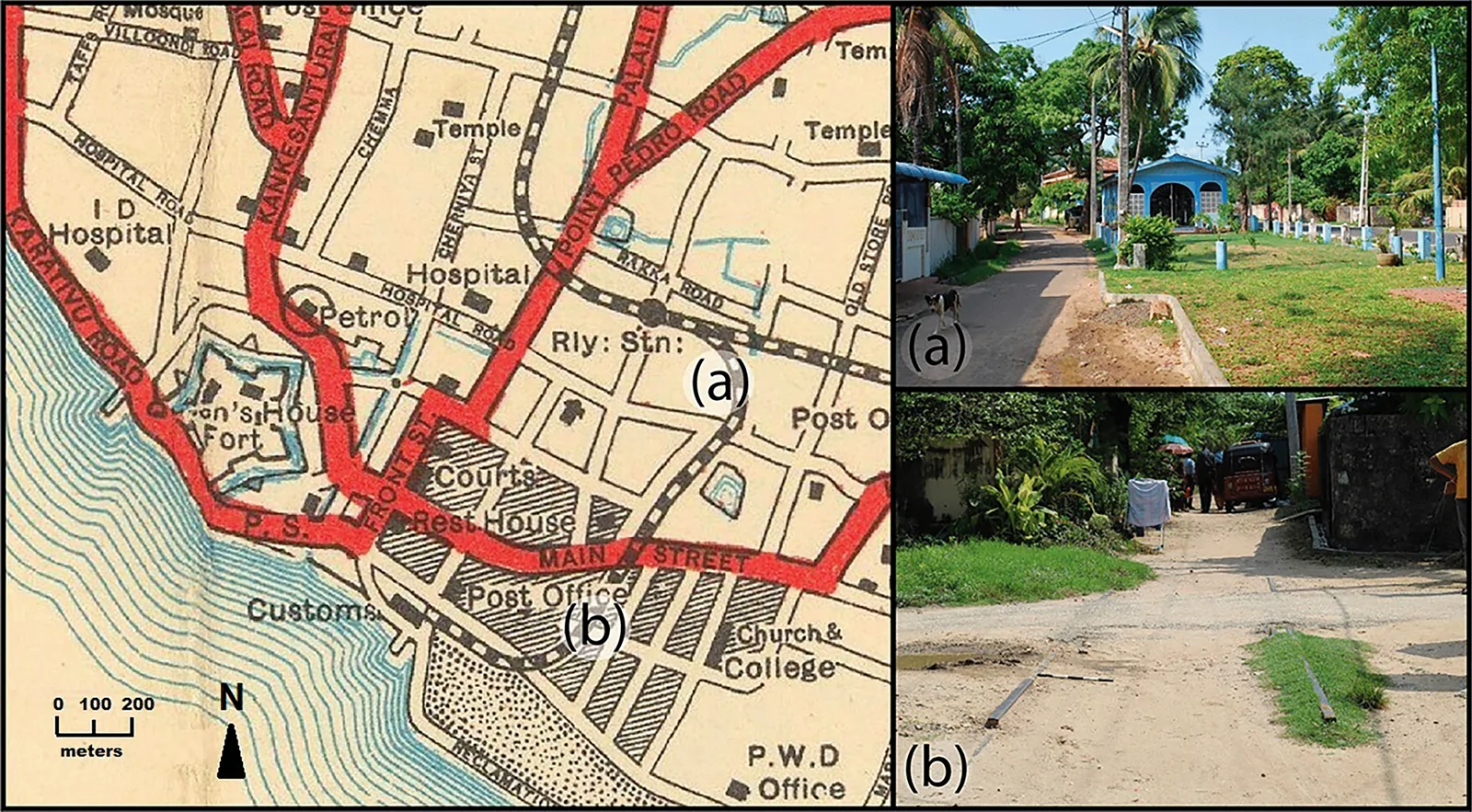
*Left*: The Jaffna branch line shown on the 1936 map (Maddams 1936); *right*: (*a*) the width of the line now occupied by a small park and church, and (*b*) remnant tracks in the surface of the narrow lane south of the main road. (Photos by authors, 2023.)

During the WHFC event of 2019 we were shown a short length of railway track embedded in the road surface at a junction on the main road into Jaffna town. Members of the Jaffna group explained this was a remnant of a branch line that had connected the main railway to the quayside. A 1936 map shows the branch line clearly (Fig. 2), and a preliminary assessment using Google Earth Pro showed that a curving line composed of roads and paths was discernible through the otherwise linear grid plan of residential streets. The track remnants were well known to local residents who had an affection for the feature. We decided that this offered a good starting point for our first walk, being within the town in terrain familiar to most participants.

Sri Lanka’s first railway line, built by the British to connect the plantations of the central highlands with Colombo port, began commercial service in 1865 (Nandasena and Wickremeratne 2017:28–30). Further lines, each intended to benefit a particular district or social group, were constructed sequentially (Thiranagama 2012:230). The Northern Railway Line, running 211 mi. (340 km) from Colombo to Kankesanthurai on Jaffna’s northern coast, passing through Jaffna town, is the longest on the island. Construction began in 1900 and was complete by 1905 (Munasinghe 1988:93–94). As an expression of Sri Lanka’s postindependence confidence, new express lines were later added from Colombo to the principal cities, including Jaffna. However, with the onset of the civil war, the railway became a symbol of state control, and the LTTE carried out attacks on the military use of the infrastructure, causing its closure in 1990. According to Jaffna colleagues, the LTTE also took up sections of the track, including this branch line (Thiranagama 2012:241).

For our walk we formed two teams, one heading north from the starting point at the Convent Road junction with Hospital Road and the other south. Interestingly, the road had been resurfaced between 2019 and 2022, and the old track at the junction was no longer visible—but could, however, be detected in the narrow lane leading south (Fig. 2). Our objectives were to trace the line of the railway and document its remnants, observe and record features and buildings in proximity, and note the uses of those buildings and the railway-line space itself.

For the team heading north, once the approximate width of the line had been discerned it was readily apparent that the space of the line had been preserved in the route of Convent Road, with buildings on either side conforming to the line. More-careful examination determined that on the east side small compounds and houses occupied a portion of the width of the line, presumably built on government-owned railway land. This ribbon of encroachment followed the length of the track along Convent Road and onto Rasavinthodham Road and ultimately along a dead-end lane curving west to where it had met the main railway line at Jaffna Station. Informal conversations between the local group and residents along the way established that the encroachment was by families of the fishing community relocating from overcrowded spaces along the quayside. This had taken place over many years, with small business enterprises established and new houses built, but no formal deeds issued. Careful examination of the path surface alongside Convent Road revealed remnants of compacted coral and limestone rubble, a feature of British-period construction, and earthwork traces of the embankment along the eastern side. Interestingly, the space of the railway line on Rasavinthodham Road has been repurposed as a playground area adjoining a small church and a minor local government office or Grama Niladhari (Fig. 2).

The railway walk proved a positive opening to fieldwork. The wealth of knowledge and understanding gathered surprised all the participants, and the archaeological practice of observation of the close range (terrain and ground surface), the middle distance (e.g., built structures), and macro landscape (visible, out of immediate sight, or mapped) was readily adopted. Systematic recording, however, was not as assiduously followed, with most of the group resorting to casual memory and smartphone photographs. Smartphone communications between group members and friends and family garnered views on the purpose of the branch line, ranging from knowledge about the transfer of goods between Jaffna and South India to the movement of goods between a large government warehouse at the northern port of Kankestanthurai to a distribution warehouse on the Jaffna waterfront. The 1936 map (Fig. 2) depicts the line as terminating at the customshouse and an area of reclaimed land extending east. It is this land that is heavily settled by the fishing community. Our inquiries demonstrate that the remnants of the railway are more than an overlooked archaeological feature. The railway’s biography includes a period of Jaffna’s history under British occupation and subsequent episodes of abandonment and encroachment and, as such, gives insights into the politicization of space linked to caste, class, and occupation.

#### Kayts Ferry Point

Our second field day was spent on Kayts, a large island west of the peninsula (Fig. 1). The town of Kayts itself is in the northern portion of the island at the strategic entry point into the lagoons of Jaffna from the deeper waters of the Palk Strait. Historically, two forts guarded the channel, both Portuguese in origin and dating from the 17th century: one at Kayts and the other at Hammenhiel, a small island between Kayts and neighboring Karaitivu Island (Nelson and R. de Silva 2004:93,96a–d). The strategic and economic importance of this location, which links the peninsula to the trading networks of the Bay of Bengal and southern India, is further underlined by the remains of a large British-period customs station. Today, there is also a Sri Lanka Navy post and a ferry point connecting Kayts with Karaitivu (Nelson and R. de Silva 2004:101b).

**Fig. 3:**
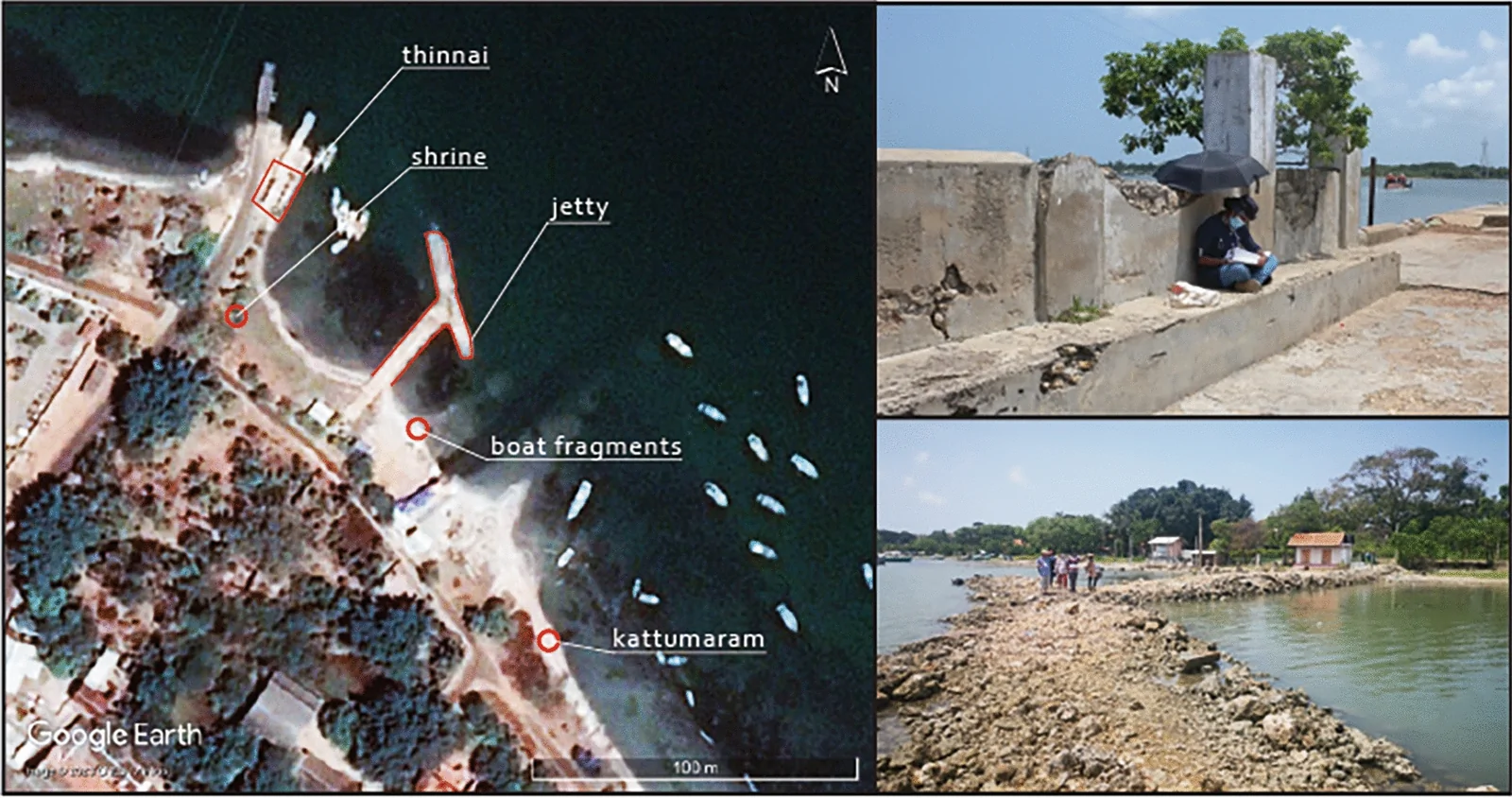
Features recorded during the Kayts field walk, including the *thinnai* (*top right*) and jetty (*bottom right*). (Map and photos by authors, 2023.)

Our focus at Kayts was the ferry point and its environs. We again divided into groups, looking at buildings and boats and mapping places of interest (Fig. 3). At the ferry point itself is a platform (*thinnai*) used by the public as a waiting place for the ferry. Although unprepossessing and scarred by war and without its traditional sheltering roof, it is nonetheless a structure of local importance and history. Under guidance from coauthor Emma Dwyer, the *thinnai* was used to introduce the basics of buildings archaeology using measured drawings and photography, and analyzing its building materials and structural stratigraphy, which showed evidence of damage from shells and bullets, as well as developing an understanding of how it fitted into the coastal landscape. These techniques helped to challenge assumptions that the group had about archaeology. Several group members had experience in the fine arts and architecture and were able to bring their skills and insights to bear.

**Fig. 4:**
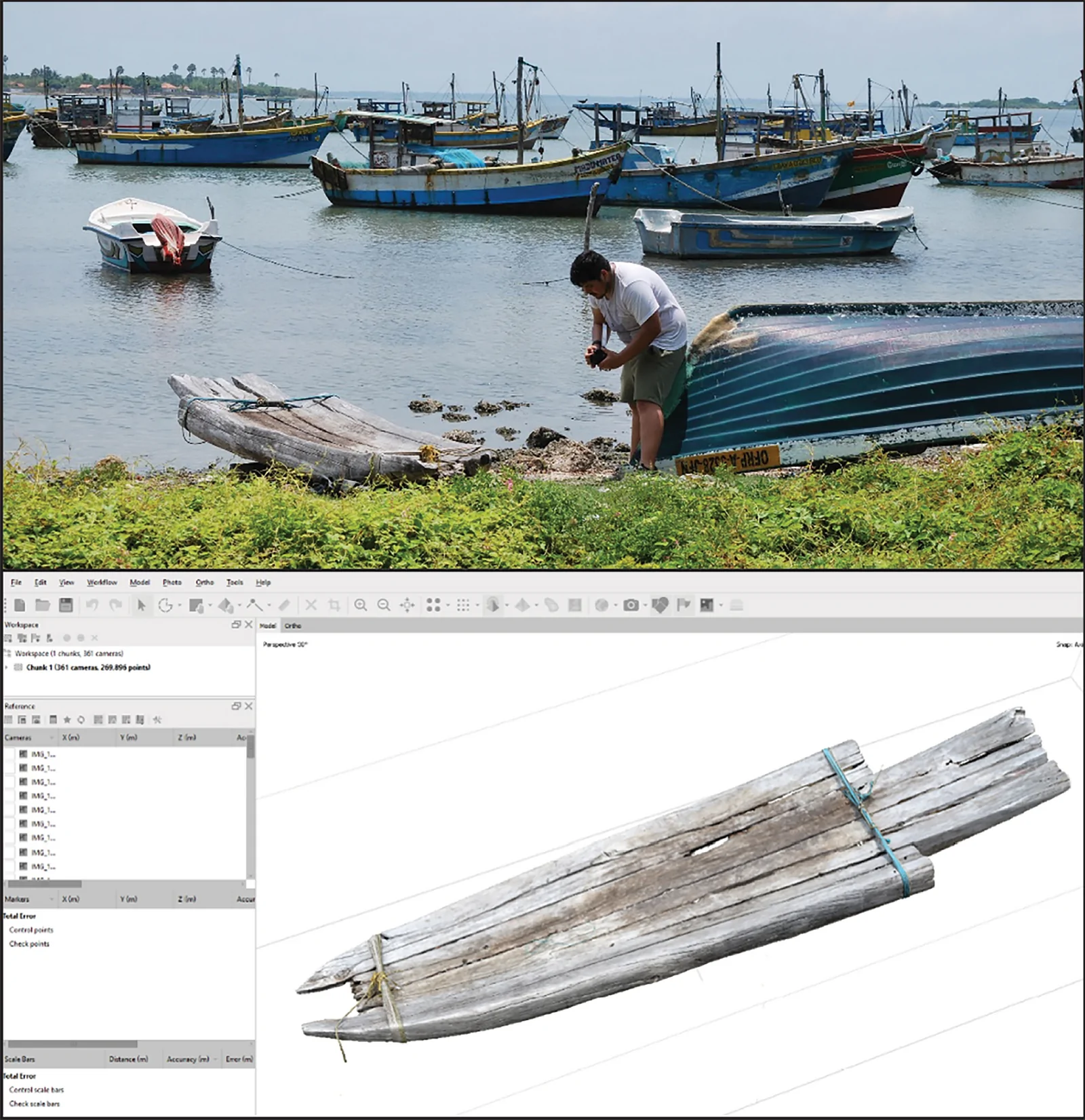
In-field photographic recording at Kayts of a traditional *kattumaram* (*top*) for motion photogrammetry (*bottom*). (Photo and image by authors, 2023.)

The boat group examined craft beached on the edge of the lagoon. A wooden *kattumaram* offered the opportunity to explore a structure using 3-D photogrammetry as a relatively rapid in-field recording method, albeit one requiring post-processing capabilities and hardware (Fig. 4). The diverse maritime niches of the Jaffna Peninsula, lagoons, sheltered inshore waters, and exposed coasts, demand diverse responses in terms of watercraft technologies, each telling a communal and individual story of cultural connection with place, including the sea. This aspect of the work drew methodologically and thematically on team member John P. Cooper’s maritime-heritage engagements elsewhere, not least Tanzania (Cooper, Blue et al. 2021; Ichumbaki et al. 2021; Cooper, Ichumbaki et al. 2022).

The group’s consideration of ways to record and map the landscape took in a broader view of what was visible within a short walk of the ferry point and of interconnections and spaces between features. Despite a large component of the 360° view from the ferry point being composed of water, there is a wealth of historical and heritage material, from the manifestations of everyday life at the *thinnai* itself, the boats, the rubble jetty, fishing paraphernalia, and a waterside Christian shrine, to the symbols of authority and control in the abandoned British customshouse and the current naval post (Fig. 3). In this instance we experimented with using a handheld global navigation satellite systems (GNSS) receiver device to geolocate the main features recorded, with data later translated into a map.

The mapping of the area adjacent to the ferry point and the surveying of the old railway line in Jaffna were collaborative recording exercises in which decisions about what elements would be included in the cartography were made collectively. This form of survey introduced participants to mapping as a tool for capturing reality from their viewpoints; it drew on the experiences of team member Adrián Oyaneder, who uses similar approaches in his work in South America. We adopted this strategy based on a critical stance, taking into account the long-lasting Western epistemic repression generated by official cartography (Harley 1989), echoing perspectives of participatory mapping and counter-mapping as emancipatory tools (Hodgson and Schroeder 2002; Sletto 2009; Berman-Arévalo 2019).

#### Point Pedro Coast

The third fieldwork day took us to the northeastern point of the peninsula, to the settlement of Point Pedro. We again focused on the land-sea interface and walked the coast to the east of the early 20th-century British lighthouse. We divided into two teams, one surveying landward structures and the other daily life along the shoreline. Consolidating the experience of the first two field days, Jaffna colleagues led the fieldwork, making decisions on what to record.

**Fig. 5:**
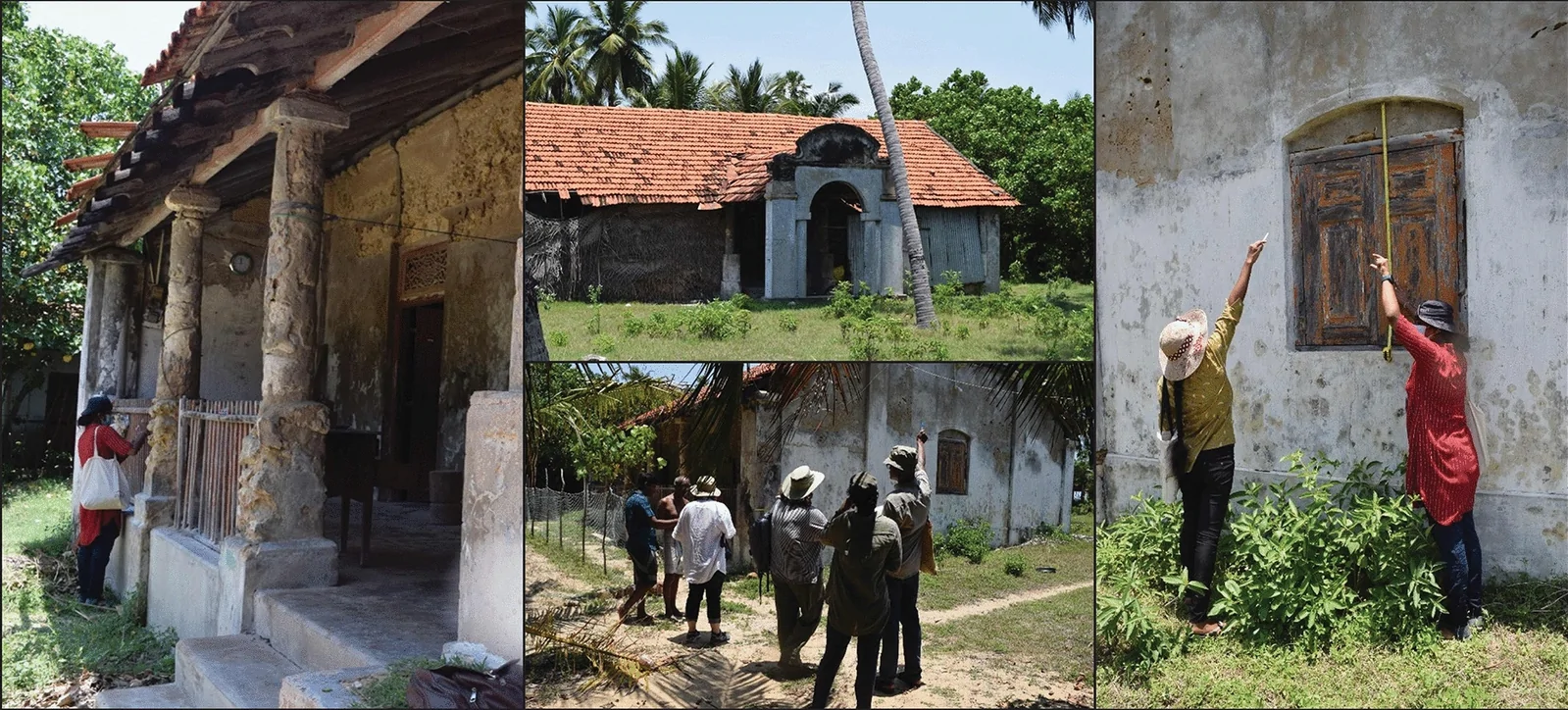
Building recording of a British-period bungalow at Point Pedro. (Photos by authors, 2023.)

Point Pedro is an affluent town, yet is an area that saw fierce resistance during the civil war. The buildings encountered were generally colonial-era bungalows with large compounds, suggesting established private ownership, although many were abandoned or partially destroyed, some having been occupied by separatists during the fighting. The team selected one house to record, producing high-quality measured drawings of the exterior. It was dilapidated and appeared vulnerable to collapse or deliberate demolition (Fig. 5). The team learned from its occupier that it belonged to a wealthy Wesleyan family, most of whom now lived abroad.

The group recording the coast spent time learning about local fishing and maritime traditions, including boatbuilding, rights of access to the water and harbor areas, and fishmongering techniques. Conversations with members of the fishing community were conducted in Tamil, led by a University of Jaffna researcher native to Point Pedro. What emerged was a realization that the sea represents a “landscape” for those living by and from it, with its own topographies of tenureship and activity. We also learned about the character of the sea: the waters immediately north are referred to as “she” or the female sea, but where the coast turns southeastward the sea changes dramatically and is gendered as male. A settled local community fishes the female sea, while itinerant groups fish the male sea according to the season. Inevitably, the craft they use and the fish they catch also change, and perceptions and uses of the sea vary markedly over a short distance.

## Impact and Insights

### Unsettling Archaeologies and Walking Together

As part of the fabric of the historical archaeology of Jaffna it was inevitable that we would encounter evidence of conflict during our fieldwork. This goes beyond the obvious physical impacts and reminders of war, such as shell damage and building abandonment, to include locations that can trigger difficult memories or that remain contentious today. How these encounters would emerge could not be anticipated, but our collective experience through the course of the workshop demonstrated the value of walking together and engaging with such material in the field. Being in the open in an informal group of different disciplinary interests enabled conversations and observations, some challenging and painful, that may not have surfaced in the more constrained setting of a seminar room or, if they had, might not have ebbed and flowed, changing and dissipating with physical movement as they did in the open air. We give two examples.

During one walk, a team member from the United Kingdom noticed an unusual construction at the roadside and asked the group what it was. Now neglected, the structure was a short, triangular-sectioned plinth, ca. 1.5 m high, with a low, decorative outer wall and possible remnants of railings. The conversation revealed it had been an LTTE martyrs’ memorial, which local teenagers had repurposed following the war to commemorate a South Indian movie star. In wartime the LTTE had erected memorials in public spaces where flags could be flown to commemorate the lives of fallen heroes (McDowell 2012; Perera 2016). The group conversation was initially subdued, in part because of prohibitions on public memorialization, but then became more curious. Why this position? Did it relate to the lives, and deaths, of people living nearby? Should we record it? Was it heritage? Gradually the conversations waned, and the group moved on. Given the restrictions on forms of local war memorialization, the initial reluctance to acknowledge this structure was understandable, but growing trust within the group overcame reticence, and the dynamics of discussion while standing and moving gave people the freedom to withdraw or distance themselves from the debate.

On another walk, we observed a small, elaborately built, and well-maintained Christian shrine serving the local fishing community. Only after completing sketch drawings, field notes, and photographs did a member of the group point out the sapling of a Bo tree (*Ficus religiosa*), a species closely associated with Buddhism (though also linked to Hinduism, Sikhism, and Jainism), growing through and damaging the perimeter wall. To the casual observer, it looked like a natural intrusion, but group members noted the proximity of a naval post and raised the possibility that the tree was a deliberate challenge by military personnel to the local religious community. The conversation circled around this as a potentially provocative action, mirroring similar actions reported across the north and east of the island. As the group moved on to explore further, the discussion also moved on. These and other instances, though brief, were valuable in many different ways. They allowed team members from the United Kingdom to learn about the war and ethnic tension without insensitive probing, and Jaffna team members with different personal experiences and political allegiances were able to express views while directing them toward an external subject rather than each other. Being in the open meant participants could join or remove themselves from the conversation without drawing attention. Based on these experiences, our future work will use field walking not simply as a conventional archaeological data-gathering method, but also to nurture safe discussions and to enable the questioning of heritage.

### Feedback and Debate

Participants in the 2022 workshop engaged fully and enthusiastically across five busy days. They brought with them their backgrounds and skills; embraced the aims, methods, and technologies being used; and shared their thoughts and reactions. Comments from the group included

1. We didn’t think archaeology included things like boats and buildings.
2. We can see how we can use the ways of recordings buildings and things like boats to understand more about other subjects such as economics.
3. We thought archaeology would be technical, needing lots of skills and specialist knowledge.
4. We’ve never had a workshop outdoors and without papers—we expected to be in class all week.

Classroom-based sessions generated wide-ranging debate. Having a mixed group composed of creative artists from the fine arts, drama, and media, as well as economists, sociologists, geographers, and historians challenged the group’s archaeologists to recognize the subjective and selective nature of what we archaeologists often like to think of as scientific and objective. The liveliest debates centered on the meaning of heritage, conceptually and especially in the Jaffna context; what “qualifies” as heritage; and who decides? For many in the group it was liberating to attach heritage meanings to their own local communities, histories, nostalgia, and lived experiences.

### Developing Community Engagement

While the workshop was successful in building the project partnership and trialling methodologies, the project still faces the challenge of engaging more widely with nonacademic groups. We consider working with communities essential to our localizing heritage aspirations, particularly as we explore ways of presenting the archaeological heritage of the recent and everyday. Developing community connections is always demanding, and a postwar context brings with it complex issues and potential barriers that cannot be ignored (Newson and Young [2026]). While heritages of conflict might offer catharsis, there is an important duty of care to consider (Dickson-Swift et al. 2008; Holtorf 2018). The workshop participants were educated and motivated and represent, to some extent, future community leaders. This bias obviously had implications for the tone and content of conversations: while many were challenging and challenged the current situation in Sri Lankan archaeology and heritage, all were respectful and aware of multiple voices. Many have shared experiences of hardship, loss, and displacement, and thus a strong sense of community. Developing effective, ethical methods of engaging with local communities as part of the bigger project will be an indirect, multistep process driven largely by local collaborators. We are also very conscious that archaeological and heritage work with local communities is not in any way a universal panacea for post-conflict societies (Newson and Young [2026]). Further, asking questions about the responsibilities that archaeologists have concerning the political and social impacts of their work is also critical (Giblin 2014; Newson and Young 2022). It could be asked whether this work should be done at all. We believe, as Giblin (2014) argues for the value of his work in Rwanda, that postcolonial archaeology can have a critical role in post-conflict situations. An increasing volume of work is paying attention to post-conflict archaeology and heritage in different parts of the world, such as the chapters on East Africa and Northern Ireland in Newson and Young (2017). This type of work debates issues around ownership of archaeology and heritage and who should benefit, as well as being more open regarding problems, challenges, and even project failures.

Given the perceptions of and attitudes toward archaeology and heritage in the north and east of Sri Lanka discussed at the start of this article, customary routes to community engagement such as publicity campaigns could potentially provoke indifference or negativity. Nonetheless, the project has already reached a growing group of Jaffna residents. In the main, this represents the friends, families, neighbors, and social networks of the workshop participants. While out walking in the field, questions about sites and places would be sent out informally on mobile phones by participants, and answers and comments would come back and be shared, generating new conversations beyond the immediate group. Similarly, discussions took place in family homes and around meal tables after field walks, and new data would be reported back the following day. In this way, we conservatively estimate that the outreach impact of the workshop was double the ca. 20 direct participants. Based on this experience, we will continue to nurture an environment of trust and open dialogue, allowing community momentum to grow organically.

The task of connecting with disadvantaged and disaffected groups nevertheless remain (Newson and Young [2026]). As a research team, we were conscious of the risks of inadvertently doing harm, as well as the possibilities of benefit. The fact that five of the present authors were, as outsiders, essentially insulated from any negative consequences of the work was self-evident and demanded both reflection and mitigation. The guiding presence of our Jaffna author (Thamotharampillai Sanathanan) in the project’s leading triumvirate was critical in the early stages of concept development, as was the development of an open research agenda that was defined more by what it intended *not* to be—*not* an archaeology of conflict or monumentality—than by what it aspired to research: the everyday, the mundane, and the local. Moreover, by adopting methodologies of engagement that were open-ended and unbounded by tight research agendas we could allow conversations to take directions preferred by the interlocutors. This did not, in itself, guarantee harm-free outcomes, but it was an important mitigation, as was the fact that Jaffna colleagues always formed the majority in any conversation, whether in the field or in workshop seminars. The fact that interviews in the field were always conducted in Tamil and mediated by Jaffna colleagues also enabled them to filter, and temper where necessary, the interventions of outside researchers. By taking a tentative approach and adopting a learning-not-teaching attitude, we avoided insensitive situations, even when informants alluded to personal trauma. At no juncture did we encounter expressions of sectarian, anti-Sinhalese bigotry: on occasion where conversation did veer into wartime memories, the narratives were largely matter-of-fact. This was in large part due to our focus on the local and our distancing from national and international discourses. We cannot, of course, report on what went unsaid. As the project progresses beyond this first workshop, we will continue to reflect on the implications and possible ramifications of our research for the well-being of communities we work with. This will not only monitor the potential of our work to inflame fragile social situations, but also consider the successes and achievements of the cautious steps we have taken so far in creating new narratives and demonstrating new ways to apply archaeological practice. That said, if ownership of the narrative *is* to rest locally—as it ought—this should not preclude the possibility that local participants may choose to take the research forward into areas of political activism. That agency is, after all, the essence of decolonized research.

## Conclusions

Heritage and archaeology can play a positive role in post-conflict situations, and, indeed, access to them is a human right (Newson and Young 2017; Chinen 2022). As we have argued, however, the reality is somewhat different in northern Sri Lanka, where archaeological heritage is largely viewed with indifference or hostility and as not relevant to people’s lived experiences. We have explored some of the reasons for this skepticism and illustrated the need for new approaches that transcend these barriers. We gave attention to the genesis of the project, arguably assisted by the longer-than-normal preparation phase imposed by global events, that gave time and space to develop relationships of trust and build a foundation for cocreation. Our response to the ancient, monumental, and elite heritage that has so far driven the nationalistic, authorized heritage narrative of the island has been to develop the concepts of localizing heritage and exploring archaeologies that are, in contrast, local, everyday, and more recent. Archaeology at this level sits at the interface between material culture and memory. It has potential to engage a multiplicity of communities and produce outcomes that are accessible and beneficial well beyond academia.

During our 2022 workshop, we trialed these concepts and methodologies with a multidisciplinary team of colleagues from the University of Jaffna. Together we were able to demonstrate that archaeology can be practiced by nonspecialists using a place-based format to situate different forms of archaeology within a landscape-biography framework. What also emerged during the workshop was the power of research based on walking together, especially in understanding the multiple meanings embedded in difficult contemporary archaeologies of war. Working with colleagues from disciplines other than archaeology and history has helped us move from a conventional adherence to “proper,” objective archaeological recording to an appreciation of differing perspectives on and intellectual and emotional responses to local character and identity. We have also shown, collectively, that archaeology can be a hugely enjoyable and positive activity. All of this experience will be taken into future phases of the project. We reassert our consciousness of the many challenges that any heritage work in post-conflict regions faces, such as the ethics of dealing with war memories, and will continue to develop reflexive approaches in future.

Our challenge remains gaining the trust and participation of local communities beyond those centered on the university. This will be at the core of our future collaboration, with the lead taken by those based in Jaffna. Working with non-archaeologists has been a first step in this process. One route into wider engagement may be demonstrating that archaeologies of the recent past can help identify and communicate contemporary issues for communities under pressure and do so in a manner that is not adversarial. For example, contemporary archaeologies of maritime life can highlight problems of coastal overdevelopment, erosion, or dwindling fish stocks. Similarly, buildings archaeology can help identify changing patterns in social structures, lifestyles, and uses of resources. The aim of this workshop and the wider project being developed is to show that archaeology and heritage can offer forums through which lived and remembered experience of the day to day at personal and community levels can be shared and valued, and that they are not just tools of the majority. Whether in that process archaeology and heritage also advance agendas of legitimate existence, social justice, and the validity of grassroots voices, then they too might be rehabilitated as disciplines capable of healing, rather than harming.

## Footnotes

1 In addition to the 1956 “Sinhala Only” policy, some of the underlying causes of polarization include (1) Education: Policies such as standardization in university admissions in the 1970s and 1980s disproportionately affected Tamil students. Tamil youth felt disadvantaged and believed their educational opportunities were limited due to these policies. (2) Citizenship: The Citizenship Act of 1948 and amendments in the 1960s disenfranchised and marginalized Indian Tamil plantation workers, who had been brought to Sri Lanka during British rule. This created a sense of alienation and discrimination among the Tamil community. (3) Land: Land-related policies such as state-sponsored colonization programs were perceived as favoring Sinhalese settlers and altering the demographic composition in Tamil-majority areas. This led to a sense of insecurity and displacement among the Tamil community. (4) Political representation: The perception of unequal political representation and marginalization of Tamil political aspirations fueled tensions. Tamil political parties called for greater autonomy and federalism, which were not adequately addressed by the majority Sinhalese-led governments. (5) Violent incidents: The outbreak of communal violence in various instances, including the 1958 riots, 1977 riots, and 1983 anti-Tamil pogrom, further deepened the divide between the Sinhalese and Tamil communities. These incidents resulted in loss of lives, displacement, and heightened mistrust.

2 See, for example, Harris (2019) on the military’s control of Kantharodai.
